# Prevalence of High Risk HPV in HIV-Infected Women From Belém, Pará, Amazon Region of Brazil: A Cross-Sectional Study

**DOI:** 10.3389/fpubh.2021.649152

**Published:** 2021-04-29

**Authors:** Jacqueline Cortinhas Monteiro, Ricardo Roberto de Souza Fonseca, Tuane Carolina de Sousa Ferreira, Luana Lorena Silva Rodrigues, Andreza Reis Brasil da Silva, Samara Tatielle Gomes, Rodrigo Vellasco Duarte Silvestre, Andréa Nazaré Monteiro Rangel Silva, Ilze Pamplona, Antonio Carlos Rosário Vallinoto, Ricardo Ishak, Luiz Fernando Almeida Machado

**Affiliations:** ^1^Biology of Infectious and Parasitic Agents Post-Graduate Program, Federal University of Pará, Belém, Brazil; ^2^Virology Laboratory, Institute of Biological Sciences, Federal University of Pará, Belém, Brazil; ^3^Papillomavirus Laboratory, Evandro Chagas Institute, Health Ministry of Brazil, Ananindeua, Brazil; ^4^Reference Unit Specialized in Infectious and Parasitic Diseases, Belém, Brazil

**Keywords:** HPV, HIV/AIDS, sexually transmitted disease, epidemiology, amazon region

## Abstract

Human papillomavirus (HPV) is the most common sexually transmitted infection in the world. Several studies have shown a higher prevalence of HPV infection in HIV-infected women. The aim of this study was to determine the prevalence and the genotype diversity of HPV infection in HIV-infected women. From April 2010 to December 2012 cervical specimens were collected from 169 HIV-infected women who screening for cervical cancer at Reference Unit in Belém. The detection of HPV infection was performed by nested PCR and HPV type was performed using a commercial system. The prevalence of HPV infection was 63.3%. Of the 47 genotyped samples, 40.4% was found positive for high risk-HPV 16 and 12.8% for high risk-HPV 52. HPV infection was predominant in the group of women with no incidence of cytological abnormalities and more prevalent in women of reproductive age, unmarried, low education level, and who reported use condoms during sexual intercourse. It was observed an association between HPV infection and independent variables, such as condom use, multiple sexual partners, and history of sexually transmitted diseases. High-risk types of HPV infection were prevalent in our study. Infection with multiple high-risk HPV genotypes may potentiate the development of cervical cancer in HIV-infected women.

## Introduction

The human papillomavirus (HPV) belongs to the Pappillomaviridae family, which currently consists of two subfamilies that include more than 50 genera and more than 130 species that can infect various classes of vertebrates (1). Morphologically, papillomaviruses are non-enveloped viruses, ~55 nm in diameter. The capsid exhibits icosahedral symmetry and surrounds the viral genome, a double-stranded circular DNA molecule of ~8,000 nucleotide base pairs (bp). Thus far, 228 HPV genotypes have been identified (2).

HPV infects the epithelial surface and can lead to the development of proliferative benign lesions in the skin, mucosa and genital tract (3). Genital HPV infection is among the sexually transmitted infections (STIs) with the highest incidence and prevalence worldwide (4–7) and is associated with the development of low- to high-grade squamous intraepithelial lesions (LSIL and HSIL, respectively).

Currently, the World Health Organization pinpoints HPV as the causal agent of cervical cancer, and virus types are classified as high- and low-risk according to their oncogenic potential. The most common low-risk HPV types are HPV-6 and HPV-11, which are most frequently detected in benign genital warts (8, 9). Among the most prominent high-risk types, HPV-16, HPV-18, HPV-31, and HPV-45 are frequently found in squamous cell carcinomas of the cervix, accounting for almost 80% of cases (10) HPV-16 and HPV-18 are responsible for 50 and 20% of all cases worldwide, respectively (11–15).

The prevalence of HPV infection can vary significantly according to the studied population and HPV detection methods. High rates have been described among sexually active adolescent girls and human immunodeficiency virus (HIV)-positive women (16–21). In immunocompetent women, HPV infection resolves within up to 24 months. However, ~10% of affected women develop a persistent infection (22). The high rate of HPV prevalence among HIV-infected women is thought to be due to the compromised immune system caused by HIV infection, thus enabling viral persistence of HPV (23) and increasing the probability of contracting infections from multiple HPV genotypes, resulting in a higher risk of progression to cervical neoplasia (24, 25).

The HPV types related with the development of HSIL and cervical intraepithelial neoplasia (CIN) in HIV-positive women living in Pará state, in oriental Amazon Region are not well-characterized. In this sense, the present study aimed to describe the prevalence of infection by different HPV types among HIV-positive women in the city of Belém, Pará State, Brazil.

## Materials and Methods

### Type of Study and Ethical Aspects

The present work is an observational population-based cross-sectional prevalence study in which epidemiological information was obtained from a single data collection. Abiding by resolutions 196/96 and 347/05 of the National Health Council, the present project was submitted to review and was approved by the Research Ethics Committee of the Instituto de Ciências da Saúde of the Universidade Federal do Pará—UFPA, under the protocol number 1765/10.

### Ethics

Written informed consent were obtained from all 169 women for the publication of any potentially identifiable images or data included in this article.

### Study Population

**Figure 1:**
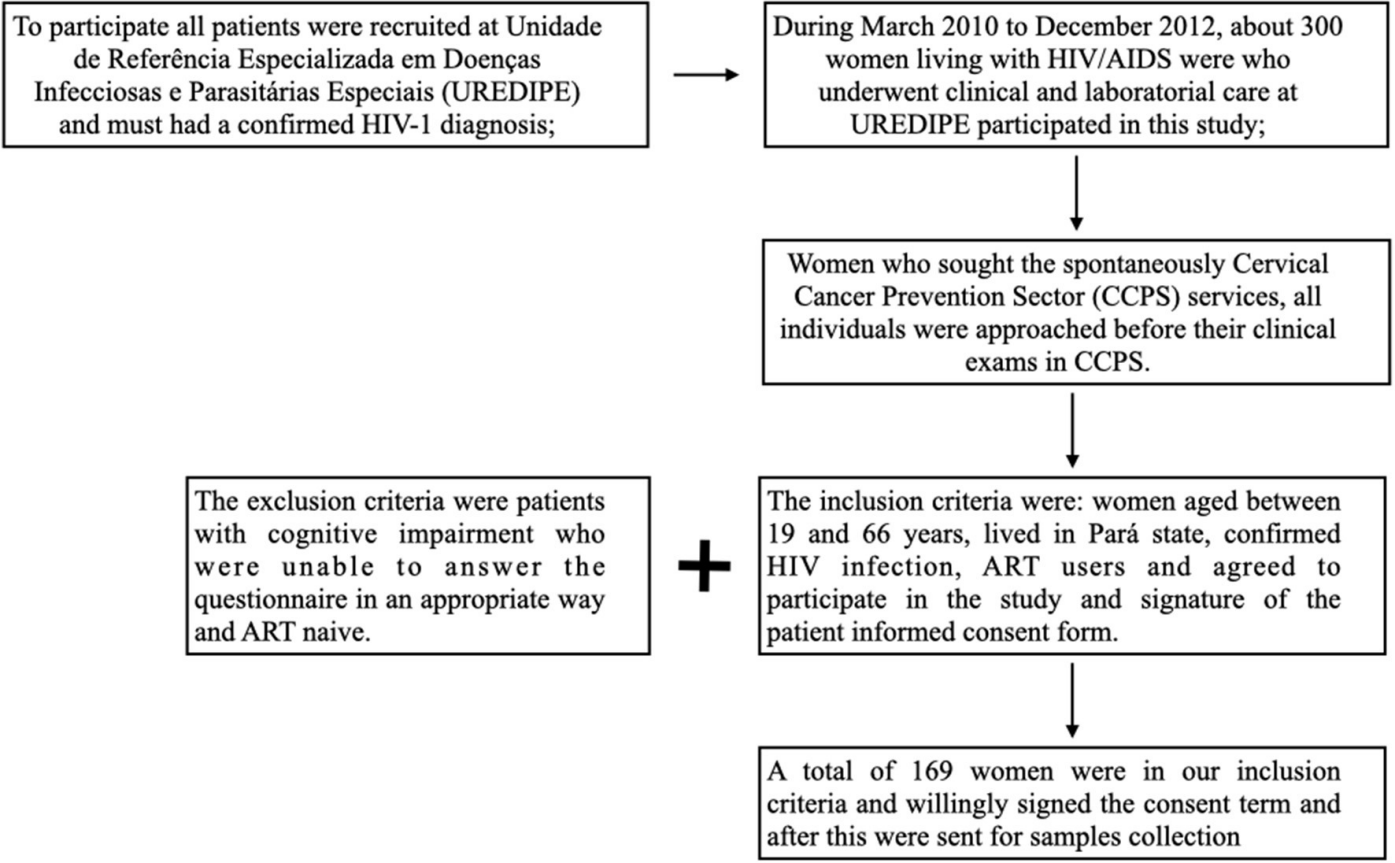
Flowchart for the selection of study participants.

A total of 169 women with a previously confirmed diagnosis of HIV-1 infection who underwent clinical and laboratorial care at Unidade de Referência Especializada em Doenças Infecciosas e Parasitárias Especiais (UREDIPE) under the Executive Secretariat of Public Health of the State of Pará (Secretaria Executiva de Saúde Pública do Estado do Pará—SESPA) participated in the study from March 2010 to December 2012 (Figure 1). The UREDIPE is a reference center that provides HIV services and care for HIV within the public health system in city of Belém, Pará, Brazil. Among the HIV services and cares provided there is the Cervical Cancer Prevention Sector (CCPS) and all individuals who participated this study sought spontaneously CCPS services.

The inclusion criteria were: women aged between 19 and 66 years, lived in Pará state, confirmed HIV infection, ART users and agreed to participate in the study and signature of the patient informed consent form. The exclusion criteria were patients with cognitive impairment who were unable to answer the questionnaire in an appropriate way and ART naive.

### Cytological Samples and DNA Extraction

Two uterine cervix smears samples were collected from each participant: one was processed on slides for cytological analysis according to the guidelines for cervical cancer screening of the Ministry of Health of Brazil, and the other was submitted to molecular biological analysis for HPV testing.

Slides were stained by the Papanicolaou method and were analyzed by a professional specializing in cytopathology at the Laboratório Central do Estado do Pará (LACEN-PA). Cytology results were classified according to the Brazilian Nomenclature for Cervical Cytology Reports (26). Descriptive diagnosis involves normal cytological limits of the examined material, the occurrence of benign alterations (inflammation, repair, immature squamous metaplasia, inflammatory atrophy, radiation) and the occurrence of pre-malignant or malignant alterations (atypia of undetermined significance—squamous, glandular, and of undetermined origin; LSIL; HSIL; carcinoma *in situ*).

Uterine cervix samples intended for molecular biological analysis were collected with endocervical brushes and placed in flasks containing 2 mL of phosphate-buffered saline (PBS). After collection, samples were taken to the Virology Laboratory of the Institute of Biological Sciences of the UFPA, where they were registered and stored at −0°C until use.

All samples were submitted to total DNA extraction from exfoliated cervical cells (cervix sample) by means of phenol-chloroform extraction (27).

### Nested PCR

The HPV L1 open reading frame (ORF) (450 bp) was amplified by means of nested-PCR with primers MY09/11 (first stage) and GP05+/06+ (second stage), which were previously described (28, 29). Each reaction contained a final volume of 50 μL with 200 ng of extracted DNA; 200 μM of each dNTP; 10 pmol of each primer; 50 nM KCL, 2.5 mM MgCl2; 10 mM Tris-HCl, pH 8.3, and 1 U of Taq polymerase (29).

All amplifications were performed in a Peltier Thermal Cycler (Biocycler, USA) using the following settings: 95°C for 5 min, 40 cycles of (95°C for 1 min; 56°C for 1 min; 72°C for 1 min), and a final extension at 72°C for 10 min. PCR products were submitted to electrophoresis (100 V/45 min) in 2 % (HPV) and 3 % (interleukin genes) agarose gel with 1x TAE buffer containing 6 μL of SYBR Safe (Invitrogen, Oregon, USA) and were then visualized in a UV transilluminator.

### Genotyping

HPV genotyping was performed with the Linear Array HPV Genotyping Test (Roche Molecular Systems, Inc., New Jersey, USA) according to the manufacturer's instructions. This method allows the detection of 37 different types of HPV.

Information on CD4^+^/CD8^+^ T lymphocyte counts and plasma HIV-1 viral load was obtained by means of access to the Laboratory Test Control System of the Brazilian National CD4^+^/CD8^+^ T Lymphocyte Count and Viral Load Network.

### Statistical Analysis

The results on HPV prevalence in HIV-1-infected patients were correlated with epidemiological information obtained from interviews using Chi-square test, G-test and Exact Fisher's Test.

## Results

The global prevalence of HPV infection in the studied population was 63.3% (107/169). Epidemiological profile analysis of the group of HIV-positive women co-infected with HPV revealed that infection was most prevalent among women of reproductive age (64.5%; 69/107); between 19 and 38 years; single, separated or widowed (55.1%; 59/107); and with a low education level (52.3%; 56/107). Furthermore, prevalence decreased with increasing age, with a 1.6 odds ratio of infection in the group of women of reproductive age.

There were no statistically significant relationships between HPV infection and the variables age, marital status and education level. There were also no statistically significant relationships with the risk factors for infection by HPV (drinking, smoking, and illegal drug use), given that the highest prevalence of infection was found in the group that exhibited none of these habits (Table 1).

**Table 1:** Demographic characteristics of women co-infected with HPV/HIV who were seen at the UREDIPE in the period between April 2010 and December 2012.

**Demographic variables**	**HIV+/HPV (+)**		**HIV+/HPV (–)**		**OR^**a**^**	***P***
	***N***	**%**	***N***	**%**		
**Age**					1.6	0.2465
19–28 years	26	24.3	08	12.9		
29–38 years	43	40.2	28	45.2		
39–48 years	23	21.5	18	29.0		
49–58 years	05	4.7	07	11.3		
> 59 years	04	3.7	01	1.6		
Not informed^*^	06	5.6	–			
Total	107	100	62	100		
**Marital status**					1.3	0.2692
Single/separated/ widowed	59	55.1	31	50.0		
Married/stable union	46	43.0	31	50.0		
Not informed^*^	02	1.9	–			
Total	107	100	62	100		
**Education level**					1.3	0.4240
≤ 8 years	56	52.3	28	45.2		
> 8 years	50	46.7	34	54.8		
Not informed^*^	01	1.0	–	–		
Total	107	100	62	100		
**Drinking**					0.7	0.2953
Yes	41	38.3	29	46.8		
No	65	60.7	31	50.0		
Not informed^*^	01	1.0	02	3.2		
Total	107	100	62	100		
**Smoking**						
Yes	24	22.4	12	19.4	1.2	0.7996
No	82	76.6	49	79.0		
Not informed^*^	01	1.0	01	1.6		
Total	107	100	62	100		
**Illegal drugs**					1.8	0.4911
Yes	11	10.3	04	6.5		
No	86	80.4	56	90.3		
Not informed^*^	10	9.3	02	3.2		
Total	107	100	62	100		

* *Not included in statistical analysis*.

a *Odds ratio*.

With respect to sexual behavior, approximately half the HPV-infected women (50.4%) had their first sexual intercourse at the age of 15 years. Most self-reported to be heterosexual (96.2%; 103/107), with an active sex life (76.6%; 72/107), in a steady relationship (69.2%; 74/107) and to practice anal sex (46.7%; 50/107). There were statistically significant associations between infection and the use of condoms and with the number of different sex partners in the past year, with a 2.6-fold higher chance of infection in the group of women who regularly used condoms during sexual intercourse (Table 2).

**Table 2:** Distribution of sexual behavior variables in a group of women co-infected with HPV/HIV who were seen at the UREDIPE and who had Pap smears in the period between April 2010 and December 2012.

**Variables**	**HIV+/HPV (+)**		**HIV+/HPV (–)**		**OR^**a**^**	***P***
	***N***	**%**	***N***	**%**		
**Age of first sexual intercourse**					1.2	0.5961
≤ 15 years	52	48.6	27	43.5		
> 15 years	54	50.4	35	56.5		
Not informed^*^	01	1.0	–	–		
Total	107	100	62	100		
**Sexual orientation**						
Heterosexual	103	96.2	61	98.4	0.6	0.9801
Homosexual	01	1.0	01	1.6		
Bisexual	02	1.8	–	–		
Not informed^*^	01	1.0	–	–		
Total	107	100	62	100		
**Sexually active**						
Yes	82	76.6	43	69.4	1.5	0.3351
No	24	22.4	19	30.6		
Not informed^*^	01	1.0	–	–		
Total	107	100	62	100		
**Steady relationship**						
Yes	74	69.2	39	62.9	1.4	0.3788
No	26	24.3	20	32.3		
Not informed^*^	07	6.5	03	4.8		
Total	107	100	62	100		
**Use of condom**						
Regular use	55	51.4	19	30.6	2.6	0.0079
Irregular use/does not use	43	40.2	39	62.9		
Not informed^*^	09	8.4	04	6.5		
Total	107	100	62	100		
**Anal Sex**						
Yes	50	46.7	25	40.3	0.7	0.4827
No	43	40.2	29	46.8		
Not informed^*^	14	13.1	08	12.9		
Total	107	100	62	100		
**Partners n°/past year**					0.2	0.0005
1	47	55.2	33	92.1		
>2	40	44.8	05	7.9		
Total	87	100	38	100		

* *Not included in statistical analysis*.

a *Odds ratio*.

The occurrence of HPV infection was highest in the group of women with a previous history of STIs (68.2%; 73/107), no history of genital warts (65.4%; 70/107), and on antiretroviral therapy (ART). The chance of acquiring an HPV infection was 17.6-fold higher in the group with a history of STIs and 2-fold higher in the group with a history of genital warts (Table 3).

**Table 3:** Distribution of the variables history of STI and genital warts in a group of women co-infected with HPV/HIV who were seen at the UREDIPE and who had Pap smears in the period of April 2010 to December 2012.

**Variables**	**HIV+/HPV (+)**		**HIV+/HPV (–)**		**OR^**a**^**	***P***
	***N***	**%**	***N***	**%**		
**History of STI**						
Yes	73	68.2	07	11.3	17.6	<0.0001
No	32	29.9	54	87.1		
Not informed^*^	02	1.9	01	1.6		
Total	107	100	62	100		
**History of genital warts**					2.0	0.2174
Yes	23	21.5	07	11.3		
No	70	65.4	42	67.7		
Not informed^*^	14	13.1	13	21.0		
Total	107	100	62	100		

* *Not included in statistical analysis*.

a *Odds ratio*.

With respect to cytology, two samples were unsuitable for analysis. Only 3.5% (6/169) of the examined cervical smears were free of cellular alterations. Furthermore, 75.8% (128/169) exhibited some type of benign alteration, and 19.5% (33/169) exhibited pre-malignant alterations. There was no statistically significant difference between cytology results and HPV infection (Table 4).

**Table 4:** Prevalence of HPV infection with respect to the cytology report in a group of HIV-infected women who were seen at the UREDIPE and who had Pap smears in the period of April 2010 to December 2012.

**Cytology**	**Overall**	**HPV (+)**	**HPV (–)**	***P***
**Normal**	06 (3.5%)	05 (83.3%)	01 (16.7%)	0.2382^a^
**Benign alterations**	128 (75.8%)	77 (60.2%)	51 (39.8%)	
Inflammatory + microorganisms	90 (70.3%)	61 (67.8%)	29 (32.2%)	**0.0099**^**b**^
Non-specific inflammatory	38 (29.7%)	16 (42.1%)	22 (57.9%)	
**Pre-malignant alterations**	33 (19.5%)	24 (72.7%)	09 (27.3%)	
ASC-US	04 (12.1%)	03 (75.0%)	01 (25.0%)	0.8420^a^
LSIL	13 (39.4%)	10 (76.9%)	03 (23.1%)	
HSIL	15 (45.5%)	10 (66.7%)	05 (33.35)	
CA	01 (3.0%)	01 (100%)	–	
Unsuitable for analysis^*^	02 (1.2%)	01 (50.0%)	01 (50.0%)	
Total	169	107	62	

* *Not included in statistical analysis*.

a *G-Test*.

b *Fisher's exact test*.

Among women with normal cytology, HPV was present in 83.3% (5/6) of the examined material. In the group of women with cytological alterations, HPV was detected in 60.2% (77/128) of the cases with benign alterations and in 72.7% (24/33) of those with pre-malignant alterations (Table 4).

In the group of women with benign alterations (128/169), inflammatory alterations associated with microorganisms corresponded to 70.3% (90/128) of the cases, among which HPV was detected in 61 samples (67.8%). With respect to samples with a non-specific inflammatory smear (29.7%; 38/128), HPV was detected in 16 specimens (42.1%).

The analysis of the type of benign alteration identified in the studied sample exhibited a statistically significant association with HPV infection, with a 2.9-fold higher odds ratio in the group that exhibited alterations associated with the presence of microorganisms (Table 4).

Among the inflammatory alterations associated with the presence of microorganisms (*n* = 90), 40% (36/90) corresponded to microbiological findings (*Lactobacillus*, cocci and other bacilli). Bacilli suggestive of infections by *Gardnerella vaginalis* were found in 32.2% (29/90) of the examined women. The presence of *Trichomonas vaginalis* and findings suggestive of HPV infection were observed in 6% of the cases.

With respect to pre-malignant alterations, 28 (84.8%) of the 33 samples exhibited intraepithelial lesions, among which 15 (45.4%) were HSIL and 13 (39.4%) were LSIL. The occurrence of atypical squamous cells of undetermined significance (ASC-US) was identified in 12.1% (6/33) of the samples. One case of invasive carcinoma (Ca) was also detected. However, there was no statistically significant relationship between the different grades of intraepithelial lesion and the presence of HPV (*G* = 0.3439; *p* = 0.8420; Table 4).

Genotyping identified the HPV types in as few as 43.9% (47/107) of the samples. Twenty-six different types were identified, among which the most prevalent were HPV-16 (40.4%; 19/47), HPV-52 (12.8%; 6/47), and HPV-84 (8.5%; 4/47).

Furthermore, among the genotyped samples, 51.0% (24/47) exhibited infection by a single genotype, among which HPV-16 was found in 37.5% (9/24), followed by HPV-61 in 12.5 % (3/24). Infections by multiple genotypes corresponded to 49% (23/47), among which HPV-16 and HPV-52 were detected in 43.5% (10/23) and 21.7% (5/23) of cases, respectively (Table 5).

**Table 5:** Distribution of HPV genotypes identified in a group of women infected with multiple genotypes and co-infected with HIV who were seen at the UREDIPE in the period of April 2010 to December 2012.

**Sample**	**Genotype**	**Sample**	**Genotype**
22417	HPV-16^*^ and 61	23292	HPV-52^*^, 53, 61 and 70
22435	HPV-6, 72 and 81	23293	HPV-16^*^, 52^*^, 62 and 83
22444	HPV-72, 81 and CP6108	23307	HPV-16^*^ and 45^*^
22447	HPV-16^*^ and 52^*^	23325	HPV-83 and 69
22456	HPV-16^*^, 52^*^ and 61	23345	HPV-16^*^ and 72
22485	HPV-16^*^, 35^*^ and 52	23455	HPV-54 and 71
22517	HPV-16^*^, 58^*^, CP6108	23459	HPV-31^*^ and 81
22520	HPV-51^*^, 66 and 83	23541	HPV-18^*^, 81 and 84
22526	HPV-58^*^, 83 and CP6108	23545	HPV-16^*^, 73^*^ and 84
22527	HPV-52^*^, 62 and 83	23547	HPV-59^*^ and 62
22556	HPV-45^*^ and 70	23574	HPV-18^*^ and 51^*^
22557	HPV-16^*^, 59^*^ and 61		

* *High-risk genotype*.

The classification of species according to oncogenic potential showed that 72.3% (34/47) of the infections were high-, 21.3% low-, and 6.4% intermediate-risk genotypes. Among the samples with infections by multiple genotypes, 39.1% (9/23) exhibited more than one high-risk genotype (Table 5). There were no statistically significant differences between levels of CD4^+^ or CD8^+^ T lymphocytes, plasma HIV viral load and HPV infection.

## Discussion

In Brazil, according to data of the National Cancer Institute (30), cervical cancer is the most common cancer type together with breast cancer among women of the North Region of the country, with an estimated risk of 26,24 cases per 100 thousand women. Many studies have shown that an infection by an HPV of high oncogenic risk, especially HPV-16 and 18, is closely related with the development of cervical cancer, with a higher chance of occurrence among HIV-positive women (14).

According to studies in the literature, HPV infection occurs in the first two consecutive years after first sexual intercourse, given that the incidence is higher among women below 25 years of age (13, 17). In the present study, among the group of HPV-infected women, the mean age was of 36 years, and infection was predominant among women of reproductive age, with a trend to reduced prevalence with increasing age. Similar data have been described in other Brazilian cities (31–33). The results of the present study suggest that the reduced prevalence of infection in the group of women aged 39 years and above is due to marriage and, thus, reductions in both the number of partners and exposure to HPV.

Several epidemiological and reproductive health factors have been related with the development of cervical cancer, including HPV, smoking, genetic predisposition, number of sex partners, age of first sexual intercourse, parity, miscarriages, and age at menarche (34). With respect to HPV infection, with the exception of immunosuppression caused by HIV, the literature is controversial with regard to establishing the risk factors for the acquisition of infection.

Studies on the general population of the North Region of Brazil have found diverging epidemiological associations between the examined population groups. In the Brazilian Eastern Amazon, there were strong associations between HPV infection and marital status, the use of condoms and the number of sex partners throughout life and in the past year (14, 15). The association between HPV infection and age range has been described in women of the general population, in female inmates in the Metropolitan Region of Belém (35) and in women in the municipality of Tomé-Açu (36).

Based on the results of the present study, even the regular use of condoms does not seem to provide total protection against microbial infection, given that the condom does not fully cover the male reproductive organ, hence leaving areas potentially harboring infectious particles or subclinical lesions, in the case of HPV, exposed during sexual intercourse associated to this perception we can observe the possibility that not all patients given the true response about his real behavior inserting a trend in this analysis that we cannot measure but is prudent to consider. A history of multiple sex partners is an important risk factor for the acquisition of HPV because the higher the number of partners, drive more probability to interact with a contaminated partner (3, 35, 36).

The innate immune system is known to have many protection mechanisms in the vaginal tissue against infections by pathogens, in part by ensuring the survival of the normal vaginal microbiota, which constitutes an important factor in the production of lactic acid and hydrogen peroxide, thus inhibiting pathogen growth. The acquisition of STIs promotes an imbalance in the vaginal microbiota, altering the pH and also possibly causing lesions in the lining epithelium of the cervix and vaginal wall. These changes might contribute to the development of inflammation and pathogen penetration, including HPV (37, 38). In addition to the observed association between a previous history of STIs and HPV infection, the present study found that many women exhibited clinical features compatible with bacterial vaginosis, candidiasis and trichomoniasis, suggesting that the establishment of these infections might contribute to the acquisition and onset of HPV infection. Similar data have been reported by Grinsztejn et al. (31) in Rio de Janeiro, whereas Gonçalves et al. (36) found that age was the only variable associated with HPV infection in anogenital samples from HIV-positive women.

The global prevalence of HPV infection among HIV-1-positive women in the present study corroborates the findings of Gonçalves et al. (36) but is lower than the rates observed in other Brazilian regions (32, 39). Among immunocompetent women of the North Region, the prevalence rate of HPV infection varies between 6, 9, and 18% (35, 36, 40) showing that the incidence of HPV infection is high in the population of HIV-positive women.

The majority of HPV infections are clinically unapparent or asymptomatic in immunocompetent individuals (14, 41). However, the immunosuppression caused by HIV seems to favor infection by multiple HPV genotypes, which, if oncogenic, might contribute to the progression of intraepithelial lesions to CIN (15). According to Luque et al. (42) the presence of multiple HPV genotypes in HIV-positive women is a poor indicator of prognosis in cases of CIN. In the present study, 49% (23/47) of the studied population exhibited infections by multiple genotypes, and, of these, 39.1% exhibited more than one high-risk genotype. Some samples that were positive in the nested-PCR could not be genotyped due to the fact that samples were exhausted in the first method and did not have enough quantity to be used in the Linear Array. On the other hand, nested-PCR can really overestimate the positive amplification that is not corroborated in genotyping.

Previous studies have shown that the prevalence rates of lesions and CIN are higher among HIV-positive women compared to women from the general population (16, 18, 39, 43) HIV infection is thought to change the natural history of HPV infection, favoring higher HPV persistence, reduced lesion regression rates and, consequently, progression to high-grade, or invasive squamous intraepithelial lesions (25, 44–46).

Some studies suggest that the persistence of HPV is inversely proportional to CD4^+^ T lymphocyte count and directly proportional to HIV viral load, which can also be influenced by ART (44, 46, 47). However, there was no association between markers of HIV infection and HPV infection or between the different grades of intraepithelial lesions in the present study. These data corroborate other studies on HIV-positive women from the Southeast Region of Brazil (19, 31–33). Thus, it is suggested that ART is efficient in the maintenance of low levels of HIV replication, avoiding the abrupt depletion of CD4^+^ T lymphocyte levels. However, the effect of ART on the persistence of HPV infection has not yet been characterized in the literature due to the absence of studies on that topic (20, 21).

Our study has some limitations. As mentioned in flowchart in Figure 1, the authors invited 300 women from a reference center in Pará state, however only 169 could be recruited due to inclusion criteria, so it did not include a truly population-based design. Our sample included women who all had a prolonged ART use, had history of STI, had genital warts, had pre-malignant alterations, median age between 19 and 38 years and presented a co-infection between HIV-HPV. This study is a cross sectional design study, which allows only presentation of baseline information, and the differences with the results presented by this study from other studies might be partly explained by information bias. Indeed, the study design used does not allow differentiating statistically significant relationships between HPV infection and the variables age, marital status and education level. Another possible bias is the none correlation between known cancer risk factors as drinking, smoking and illegal drug use with infection by HPV.

The data obtained in the present study show the high prevalence of HPV infection among HIV-positive women and reinforce the notion that immunocompromised women tend to develop infections by multiple high-risk HPV genotypes. Thus, the importance of molecular diagnosis of HPV infection associated with oncotic cytology in the early detection of cases, with the aim of avoiding progression to CIN, is reinforced.

## Data Availability Statement

The raw data supporting the conclusions of this article will be made available by the authors, without undue reservation.

## Ethics Statement

This project was approved by the Research Ethics Committee of the Institute of Health Sciences of the Federal University of Pará, under protocol number 1765/10. All participants signed a consent form.

## Author Contributions

JM and LM: conceptualization. TF, LR, RF, and AS: data curation. IP, RS, and AS: investigation and methodology. RS, SG, and LM: formal analysis. AV, RI, and LM: writing-original draft. RF, AV, RI, and LM: writing-review and editing. JM and LM: project administration. All authors read and approved the final manuscript and contributed to the development of research.

## Conflict of Interest

The authors declare that the research was conducted in the absence of any commercial or financial relationships that could be construed as a potential conflict of interest.
